# Tissue-based multiphoton analysis of actomyosin and structural responses in human trabecular meshwork

**DOI:** 10.1038/srep21315

**Published:** 2016-02-17

**Authors:** Jose M. Gonzalez, Minhee K. Ko, Andrew Pouw, James C. H. Tan

**Affiliations:** 1 Doheny Eye Institute and Department of Ophthalmology, David Geffen School of Medicine at UCLA, Los Angeles, CA

## Abstract

The contractile trabecular meshwork (TM) modulates aqueous humor outflow resistance and intraocular pressure. The primary goal was to visualize and quantify human TM contractile state by analyzing actin polymerization (F-actin) by 2-photon excitation fluorescence imaging (TPEF) *in situ*. A secondary goal was to ascertain if structural extracellular matrix (ECM) configuration changed with contractility. Viable *ex vivo* human TM was incubated with latrunculin-A (Lat-A) or vehicle prior to Alexa-568-phalloidin labeling and TPEF. Quantitative image analysis was applied to 2-dimensional (2D) optical sections and 3D image reconstructions. After Lat-A exposure, (a) the F-actin network reorganized as aggregates; (b) F-actin-associated fluorescence intensity was reduced by 48.6% (mean; p = 0.007; n = 8); (c) F-actin 3D distribution was reduced by 68.9% (p = 0.040); (d) ECM pore cross-sectional area and volume were larger by 36% (p = 0.032) and 65% (p = 0.059) respectively and pores appeared more interconnected; (e) expression of type I collagen and elastin, key TM structural ECM proteins, were unaltered (p = 0.54); and (f) tissue viability was unchanged (p = 0.39) relative to vehicle controls. Thus Lat-A-induced reduction of actomyosin contractility was associated with TM porous expansion without evidence of reduced structural ECM protein expression or cellular viability. These important subcellular-level dynamics could be visualized and quantified within human tissue by TPEF.

The actomyosin system of the trabecular meshwork (TM) plays critical roles in regulating aqueous outflow resistance and intraocular pressure (IOP) and has become a target for novel cell-directed glaucoma pharmacotherapy^1^,^2^,^3^,^4^,^5^,^6^,^7^,^8^,^9^,^10^,^11^,^12^,^13^,^14^,^15^,^16^. Agents that target actin directly (e.g., latrunculin) or indirectly (e.g., Rho-kinase inhibitors) are in human clinical trials^16^ based on pre-clinical testing in cell culture, organ culture and animal models^17^,^18^. It is harder to directly observe and measure altered actomyosin dynamics in the human TM, but if achieved it could provide important insights into drug-induced cellular dynamics.

The state of actin polymerization (filamentous actin; F-actin) reflects actomyosin activity within cells and tissues. Actin polymerization may be inhibited by agents such as latrunculin-A (Lat-A), which sequesters monomeric actin to prevent actin polymerization and actomyosin contractility^3^,^4^. Resulting cellular relaxation is expected to cause expansion of the drainage tissue, decreased outflow resistance, and reduced IOP^3^,^18^, which may be exploited for glaucoma therapy^16^,^19^. These agents may be used as probes to simulate actin-modulatory therapy *in situ*, decipher the role of actomyosin activity in outflow regulation, and check for possible cell toxicity as the actin cytoskeleton plays vital roles in the cell.

Contractile cells on the extracellular matrix (ECM) of trabecular beams are uniquely positioned to modulate outflow channel dimensions by regulating tension in actin cytoskeleton networks^20^,^21^,^22^,^23^,^24^. The structural ECM of trabecular beams has been modeled in cell culture (e.g. collagen gel contraction assays)^22^,^25^, and studied by scanning and transmission electron microscopy^24^, immunohistochemistry^26^,^27^,^28^,^29^,^30^,^31^, and histology (e.g., Weigert’s resorcin-fuschin^30^). None of these techniques preserve the TM’s 3-dimensional (3D) context, making it hard to directly observe the role of cellular contractility in the TM.

We have established a tissue-based approach using 2-photon excitation fluorescence microscopy (TPEF) and deep tissue optical sectioning to visualize subcellular events in the human TM^32^,^33^,^34^. During TPEF, photon absorption is restricted to the focal plane^35^,^36^, reducing photo-bleaching and preserving excitation energy through the beam path. This permits deeper laser penetration^37^ and high-resolution visualization within intact, unsectioned tissues^38^,^39^. Structural extracellular matrix proteins of elastin and collagen may be isolated out within tissues by exploiting endogenous signatures such as autofluorescence in 2-photon tissue images^40^. Were these approaches to be adapted for clinical application, intriguing insights into clinically relevant processes affecting IOP could be gained.

Herein, we report visualization and measurement of actomyosin activity in the human TM in response to Lat-A. TPEF optical sectioning, 3-dimensional (3D) software-assisted reconstruction, and quantitative image analysis were applied to measure tissue-based F-actin as a readout of contractile state. As a secondary goal, we analyzed autofluorescent signatures of the structural ECM to ascertain putative structural responses to altered contractility.

## Results

### F-actin organization in human TM

**Figure 1:**
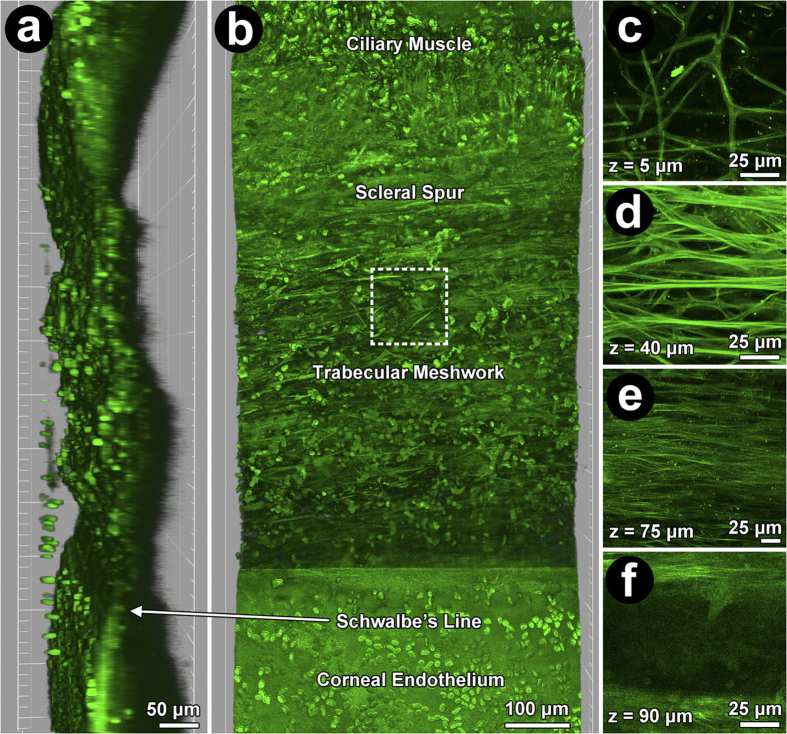
2-photon excitation fluorescence imaging (TPEF) of human trabecular meshwork (TM) showing autofluorescent extracellular matrix (ECM) and Hoechst-33342-labeled nuclei (green ovals) at low (220X) and high magnification (756X). (**a**) Orthogonal view: TM between ciliary muscle and scleral spur (posterior; top), and Schwalbe’s line and Descemet’s membrane (anterior; bottom). (**b**) En face view: TM cells and autofluorescent fibers between ciliary muscle and cornea. Dashed box: region of high magnification images (756X) of uveal (**c**; UM), corneoscleral (**d**; CSM), juxtacanalicular meshwork (**e**; JCT) and Schlemm’s canal (f; SC). (**c**): UM (mean ± SD thickness: 25 ± 14 μm) with slender branching beams separated by wide gaps (diameters >40 μm). (**d**): CSM autofluorescent plate-like beams (than UM) with smaller pores (diameters <40 μm) was 25–65 μm (~40 μm average thickness) from UM inner surface. (**e**): JCT arrays of fine autofluorescent fibers orientated parallel to SC, 65–75 μm (~10 μm average thickness) from UM inner surface. (**f**): Autofluorescence signal void of SC ~75 μm from inner UM surface.

**Figure 2:**
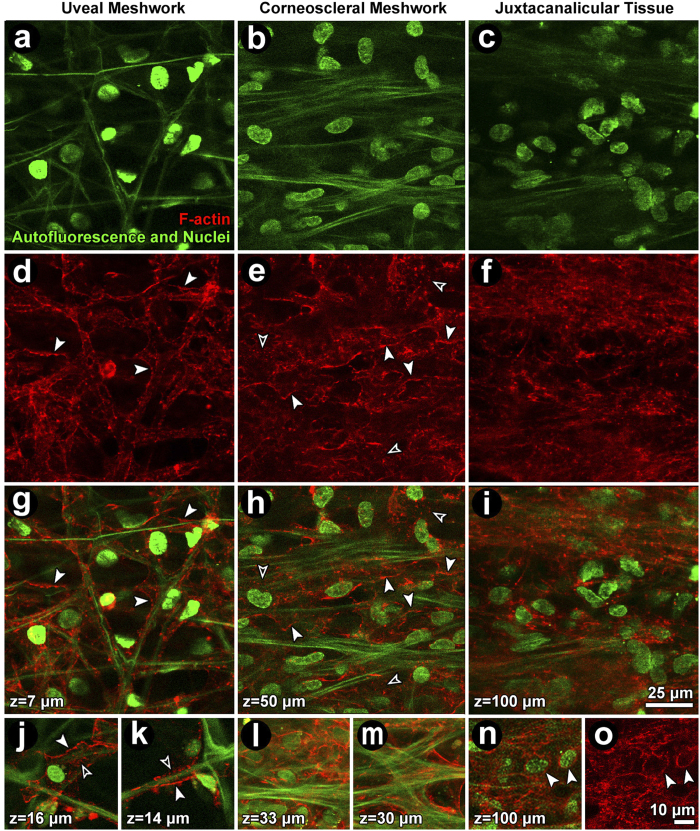
Filamentous actin (F-actin) organization in the uveal (UM; left column), corneoscleral (CSM; middle column), and juxtacanalicular meshwork (JCT; right column) of vehicle control tissue. (**a–c**) Autofluorescent structure (green fibers) and Hoechst 33342-labeled nuclei (green ovals) in UM (**a**), CSM (**b**) and JCT (**c**). (**d–f**) Cortical F-actin network in the UM (**d**), CSM (**e**) and JCT (**f**). (**g–i**) Merged images showing F-actin (red) association with autofluorescent fibers and nuclei (green). (**j–o**) Detail of F-actin in cells associated with autofluorescent structures and nuclei. *Closed arrowheads:* cortical F-actin. *Open arrowheads:* punctate F-actin. *Green:* autofluorescent structure and Hoechst-labeled nuclei. *Red:* F-actin.

The autofluorescent ECM structure of the TM provided landmarks for identifying the corneoscleral meshwork (CSM; Fig. 1d), as previously described^32^,^33^ slender autofluorescent branching beams were seen in the uveal meshwork (UM); coarser beams separated by smaller pores in the CSM; and fine fiber arrays in the juxtacanalicular meshwork (JCT) (Figs 1c–e and 2a–c).

Cortical F-actin corresponded to cell borders (Fig. 2j,k; closed arrows), forming an interconnected curvilinear network aligned with autofluorescent fibers and wrapped as a lacy network around beams (Fig. 2g–i). Punctate perinuclear F-actin distributions were also seen (Fig. 2e,h,j,k; open arrows). In the UM and CSM, F-actin wrapped around beams or spread across adjacent branching beams (Fig. 2g,h,j–m). F-actin distribution in the JCT and CSM reflected higher cellular density (Fig. 2e,f,h,i) compared with the UM. Actin stress fibers, as seen in cultured cells, were not observed in the tissue.

### F-actin reorganization

**Figure 3:**
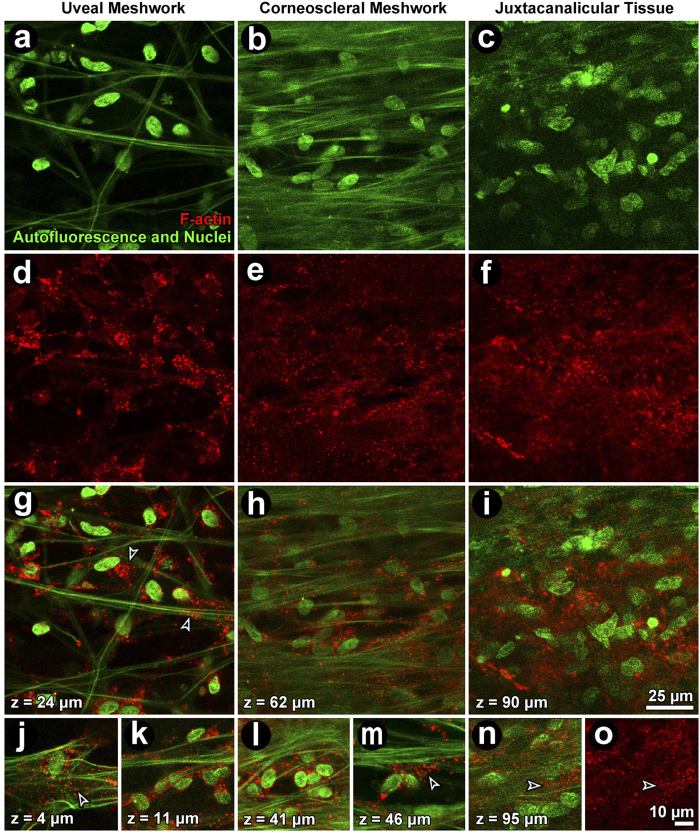
Filamentous actin (F-actin) organization in the uveal (UM; left column), corneoscleral (CSM; middle column) and juxtacanalicular meshwork (JCT; right column) after exposure to 1 μM latrunculin-A (Lat-A). (**a–c**): Autofluorescent structures (green fibers) and nuclei (green ovals) were unchanged after Lat-A treatment. (**d–f**): The F-actin cortical network in regions of the UM (**d**), CSM (**e**) and JCT (**f)** was disrupted leaving only punctate cytosolic collections. (**g–i**) (merge): cortical F-actin association with the autofluorescent structure is absent but punctate collections are prominent. (**j–o**): Detail of punctate perinuclear F-actin (red) in isolated cells in the absence of cortical actin in the UM (**j,k**), CSM (**l,m**) and JCT (n,o). *Closed arrowheads:* cortical F-actin. *Open arrowheads:* punctate F-actin. *Green:* autofluorescent structure and Hoechst-labeled nuclei. *Red:* F-actin.

Following Lat-A exposure, the prominent F-actin cortical network reorganized as aggregates (Fig. 3). This was evident in the UM (Fig. 3, left column), CSM (Fig. 3, middle column) and JCT (Fig. 3, right column). Hoechst-labeled nuclear fluorescence and autofluorescence features were unaltered in Lat-A-treated tissue (Fig. 3a–c), appearing similar to vehicle control tissue (Fig. 2a–c).

### F-actin fluorescence intensity

**Figure 4:**
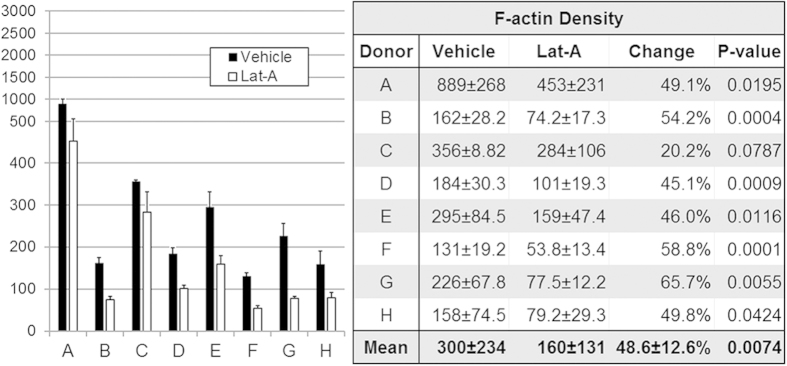
Filamentous actin (F-actin) density in vehicle control and latrunculin-A (Lat-A)-treated tissue (n = 8; **A–H**), as represented by F-actin adjusted fluorescence intensity (F-actin mean fluorescence intensity adjusted for number of cells). F-actin density was reduced by a mean of 48.6% following Lat-A exposure (p = 0.007; see table). *Black columns:* F-actin adjusted fluorescence intensity is shown for vehicle control tissue. *White columns:* 1 μM Lat-A-treated tissue. *Error bars:* standard error of the mean. *Table:* data values for mean F-actin density (±standard deviation) in vehicle control and Lat-A-treated tissue (n = 8; **A–H**). All tissues showed a trend toward reduced F-actin density after Lat-A treatment; difference was statistically significant in 7/8 tissues (87.5%). Significance testing was based on paired vehicle control and treated tissues from same eyes.

F-actin fluorescence intensity represented tissue F-actin density (Fig. 4; n = 8) in multiple regions of interest (ROI) samplings in serial optical sections through the CSM. Control and treated tissue pairs were from the same eye.

Mean (±SD) F-actin adjusted fluorescence intensity was 300.0 ± 233.6 in vehicle controls and 160.2 ± 130.5 in Lat-A-treated TM (Fig. 4 Table). This represented a mean F-actin density reduction of 48.6% ± 12.5% (p = 0.00005; n = 8) after Lat-A treatment. Significantly reduced F-actin adjusted fluorescence intensity was seen in 7/8 (88%; p < 0.05) tissues treated with Lat-A. Mean (±SD) nuclear count in ROI was similar in vehicle controls (21.2 ± 9.9) and Lat-A-treated tissue (24.4 ± 12.1; p = 0.51).

### F-actin 3D tissue distribution

**Figure 5:**
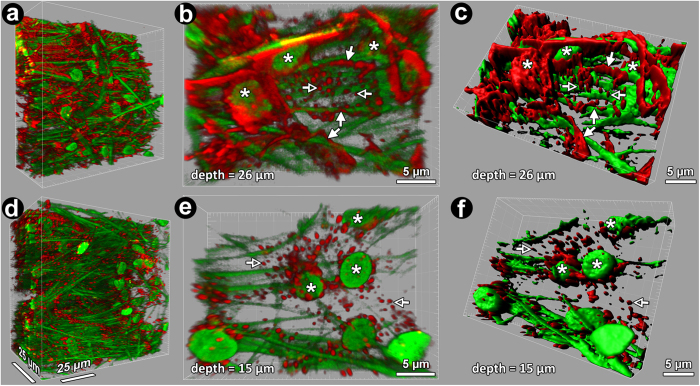
3-dimensional (3D) reconstructions of vehicle control (**a–c**) and latrunculin-A (Lat-A)-treated (**d–f**) trabecular meshwork (TM). 3D reconstructions were cropped and rotated to show *en face* and oblique tissue views. Cortical filamentous actin (red: F-actin) networks were prominent amongst nuclei and autofluorescent structures (green) in vehicle controls (**a–c**) but not in Lat-A-treated tissue (**d–f**). Punctate actin collections were prominent in Lat-A-treated tissue. **b,e**): High magnification 3D reconstruction of TM (30 × 20 × 9 μm crop of 246 × 246 × 100 μm 3D volume) showing cortical F-actin network (closed arrows) and punctate perinuclear actin collections (open arrows) associated with autofluorescent fibers and nuclei (asterisks). (**c,f**): Isosurface maps of F-actin closely correspond to phalloidin-labeled F-actin phalloidin in vehicle control (**b** vs. **c**) and Lat-A-treated tissue (**e** vs **f**), capturing treatment-induced F-actin network and punctate changes. *Asterisks:* nuclei; closed arrows: cortical F-actin network; *green fibers:* autofluorescent structure; *green ovals:* nuclei; *open arrows:* punctate F-actin collection; *red fluorescence:* F-actin.

3D actin reorganization after Lat-A treatment is depicted in 3D reconstruction and isosurface model renderings (Fig. 5). In basal state, an extensive interconnected cortical F-actin network was prominent among punctate F-actin collections that were sparse, nuclei and autofluorescent beams (Fig. 5a–c). Following Lat-A, the interconnected F-actin network was disrupted, leaving aggregates as the prominent F-actin feature (Fig. 5d–f). Supplementary 3D movies 1 and 2 provide alternative views of differences in F-actin organization in vehicle control and Lat-A-treated tissues.

Higher magnification 3D reconstructions revealed details of F-actin organization in control (Fig. 5b) and Lat-A-treated tissue (Fig. 5e). Isosurface mapping of F-actin organization and distribution in 3D space (Fig. 5c,f) recapitulated observations of labeled F-actin and was used to quantify F-actin distribution in the tissue.

**Figure 6:**
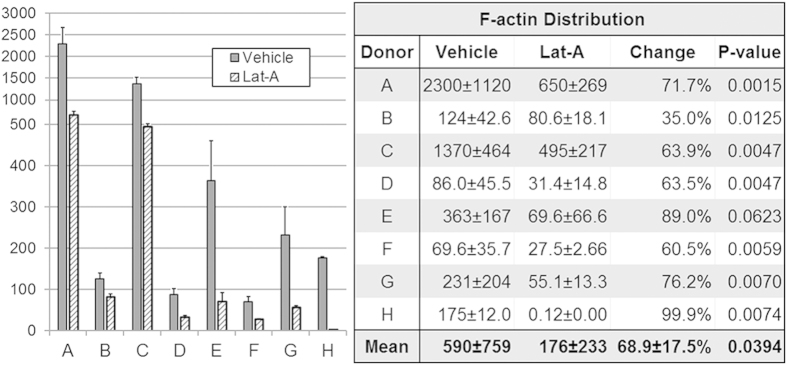
Filamentous actin (F-actin) distribution in vehicle control and latrunculin-A (Lat-A)-treated tissue. F-actin adjusted fluorescence distribution (F-actin mean adjusted fluorescence distribution adjusted for number of cells; n = 8; **A–H**) was reduced by a mean of 68.9% following Lat-A exposure (p = 0.04; see table). F-actin adjusted fluorescence distribution is shown for vehicle control tissue (dark gray columns) and 1 μM Lat-A-treated tissue (light gray hashed columns). *Error bars:* standard error of the mean. *Table:* Mean F-actin distribution (± standard deviation) in vehicle control and latrunculin-A (Lat-A)-treated tissue (n = 8; A-H), as represented by F-actin adjusted fluorescence distribution, and compared in paired vehicle control and treated tissues from the same eyes. All showed a trend to reduced F-actin distribution after Lat-A treatment; this was statistically significant in 7/8 tissues (87.5%).

Each donor eye (n = 7) contributed tissue for vehicle control and Lat-A-treated analysis. F-actin isosurface maps were analyzed in 9 tissue subvolumes per donor tissue (as illustrated in Supplementary Fig. 1C; each 82 × 82 × 38 μm) per condition (Fig. 6). Mean nuclear counts across tissue subvolumes were similar in vehicle controls (51.1 ± 21.9) and Lat-A-treated tissue (36.2 ± 18.5; p = 0.16; paired Student’s t-test). Mean F-actin adjusted fluorescence distribution in 3D space was 68.9 ± 17.5% lower in Lat-A-treated tissues (176.2 ± 233.3) compared with vehicle controls (589.5 ± 759.3; p = 0.02) (Fig. 6, Table). Significantly reduced F-actin adjusted fluorescence distribution was seen in 7/8 (88%; p < 0.05) Lat-A-treated tissues.

### Autofluorescent ECM signature

**Figure 7:**
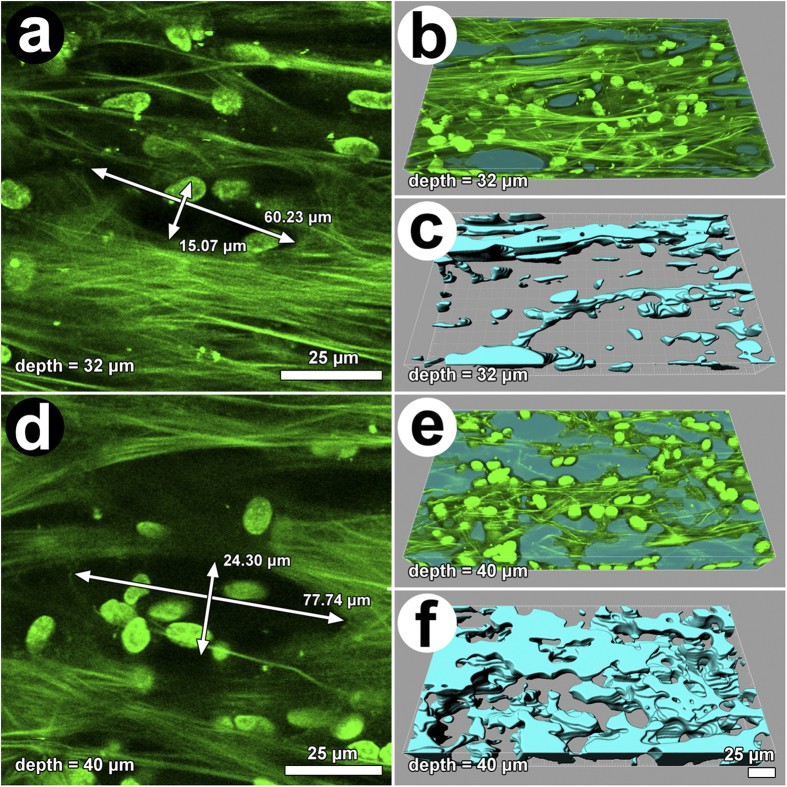
Representative autofluorescence images of the corneoscleral meshwork in basal state (**a–c**: controls) and after latrunculin-A (Lat-A) exposure (**d–f**). (**a,d**) Arrows indicate pores. (**b,c**) Dual-channel autofluorescence (green) and pore reconstruction (blue; automated software-assisted reconstruction of voids) images. (**e,f**) Isosurface mapping of tissue pores (light blue: void space) illustrating the extent to which pore dimensions increased and became interconnected following Lat-A exposure.

An organization of autofluorescent ECM beams with intervening pores was seen in the CSM, as illustrated in Fig. 7. Drainage pores appeared more prominent in tissue exposed to Lat-A (compare Fig. 7A,D). When measured in 2D optical sections, pore cross-sectional area was greater in Lat-A-treated tissue (1,103.4 ± 890.8 μm^2^) compared with untreated tissue (813.6 ± 480.0 μm^2^) (p = 0.032). In 3D tissue reconstructions, pore volumes were greater in Lat-A-treated tissue (274,042.8 ± 123,184.9 μm^3^) compared with untreated tissue (166,297.3 ± 34,933.7 μm^3^) with borderline significance (p = 0.059).

Based on ROI analysis of autofluorescent structures, Lat-A did not alter intensity of the autofluorescent signature (p = 0.54; n = 6), suggesting that the change in drainage pore size was not associated with a change of autofluorescent ECM protein content (i.e., levels of type I collagen and elastin). The average autofluorescence intensity per pixel for vehicle control specimens was 4,023.8 ± 1,366.4 and for Lat-A treated specimens was 4,486.8 ± 1,319.4 (p = 0.54).

Further specific analysis of TM extracellular levels of type I collagen and elastin by Western blot and immunohistochemistry (IHC) did not reveal differences between control and Lat-A conditions (Supplementary Figs 3 and 4; p = 0.6).

### Potential toxicity of Lat-A

**Figure 8:**
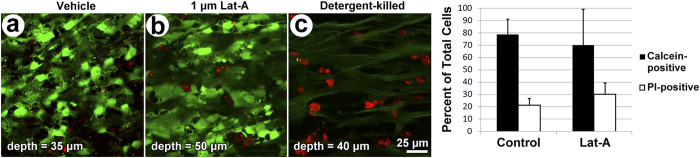
Effect of latrunculin-A (Lat-A) on cell viability and evaluation of potential toxicity. Representative optical sections of vehicle control corneoscleral meshwork (**a**; CSM), 1 μM Lat-A-treated CSM (**b**), and Triton X-100-treated CSM (**c**), which served as a positive control for toxicity-related cell death. Calcein-positive live cells (green cytosolic fluorescence) predominated in vehicle control (**a**) and Lat-A-treated tissue (**b**), whereas propidium iodide (PI)-stained nuclei of dead or apoptotic cells (red) predominated in Triton-X-treated tissue (**c**). (**d**) Quantitative toxicity analysis: column graph shows mean number of calcein-positive live cells (black columns) and PI-positive dead cells (white columns) as a percentage of total cells. 78.7 ± 12.4% (mean ± SD) of cells in vehicle control tissue and 70.0 ± 29.3% of cells in 1 μM Lat-A-treated tissue were viable (i.e., calcein-positive; black columns; p = 0.22). A mean of 21.3 ± 5.7% of cells in vehicle control tissue and 30.0 ± 9.9% of cells in Lat-A-treated tissue were viable (i.e., PI positive; white columns; p = 0.39; n = 4). *Error bars:* standard error of the mean.

Calcein AM (live) and propidium iodide (PI; dead) viability co-labeling resulted in a similar pattern in vehicle control and Lat-A-treated TM (Fig. 8a,b). Labeling patterns in both groups were different from that of detergent-killed tissue (Fig. 8c), which was used as a positive control for cell death, as previously described^41^. Calcein-positive live cells were similar in number and distribution in vehicle control (Fig. 8a) and Lat-A-treated TM (Fig. 8b), but were virtually absent in detergent-exposed tissue (Fig. 8c). PI-positive dead cells were scant in vehicle control and Lat-A-treated TM, but predominated in detergent-killed TM. Thus the vast majority of cells were alive in vehicle control and Lat-A-treated TM. Virtually all cells were dead in detergent-treated TM.

The numbers of calcein-positive cells, PI-positive cells and total cells were not significantly different between vehicle control TM and Lat-A-treated TM (Fig. 8d; p-values = 0.96, 0.34, 0.75, respectively). Mean percent live cellularity was 78.7 ± 12.4% in vehicle control TM and 70.0 ± 29.3% in Lat-A-treated TM (p = 0.39 by a manual counting method, and p = 0.35 by a software-assisted, semi-automated counting method)^41^. Mean live cellularity of untreated tissue not exposed to either vehicle or Lat-A (non-vehicle, non-Lat-A control) was 71.8 ± 12.2%^41^.

## Discussion

We have described experimental tools by which to probe and interrogate events within the TM. Here, 3D tissue relationships are preserved, unlike observations made in cultured cells, sectioned tissues or homogenized isolates.

TPEF revealed a vast interconnected network traversing cells and wrapping around TM beams. *In situ*, F-actin lacked obvious stress fibers. Following Lat-A exposure, the extensive cortical F-actin network reorganized as cytoplasmic aggregates. F-actin-associated fluorescence intensity in 2D optical sections and volumetric distribution in 3D space were markedly reduced. Associated enlargement of pores in the ECM structure was seen without evidence of compromised cell viability. Our autofluorescence, Western blot and immunohistochemistry findings agreed that porous enlargement occurred without altered content of elastin and type I collagen, which are primary contributors to the TM autofluorescence signature^40^,^42^.

Our methodologies, which may be automated^41^, are potentially useful for human tissue-based quantitative analysis with respect to tissue structural, cellular and protein signatures; we demonstrated this along the following lines: (a) effect of Lat-A on F-actin tissue density and distribution was measured in a way that accounted for variation in cell sampling across images; (b) treated and control tissues obtained from the same eye provided easy standardization without inter-individual variability; (c) multiple ROI samplings in multiple optical sections in each image series through each tissue; (d) background fluorescence did not mask detection of treatment-induced change and significant differences between treated and control tissue were easily evident; (e) isosurface mapping could include thresholding, which captured cortical actin over non-specific phalloidin labeling to exclude artifact; (f) semi-automated software-assisted measurement in 2D (e.g., pore cross-sectional area) and 3D (e.g., pore volume) tissue space was possible; (g) measurement of tissue cellular viability using live/dead intravital dye co-labeling was performed *in situ* as previously described^34^,^41^; (h) analysis was conducted in viable and inexpensive post-transplant tissue instead of whole postmortem human eyes which are scarce and costly, although the latter remains an option; (i) 2D optical sections and 3D reconstructions were not just striking images but represented vast, complex, coded data sets containing information on morphology (e.g., cellular localization; fine structure such as beams and pores), structural ECM (beams), proteins (structural collagen, elastin, actin) and protein functional state (actin polymerization representing contractility) from which we segregated and extracted specific data for analysis by computational methods; (j) we logically assembled this extracted subcellular, protein, functional and morphologic data to provide insights into tissue responses; (k) each analytical algorithm (F-actin density, F-actin distribution, structural ECM configuration, autofluorescence intensity, nuclear analysis, toxicity analysis) represented a separate mathematical approach to combine data extracted from multiple optical sections. Several aspects of this analytical approach had previously been validated^34^.

Our *in situ* observations agreed with reports using alternative methods. In electron microscopy of the TM, electron-dense actin filaments with diameters of 5–6 nm are concentrated in perinuclear and cortical regions of cells^17^. In cell culture and standard immunohistochemistry, F-actin is distributed in peripheral and perinuclear regions of TM cells, wrapped around trabeculae, and sometimes organized as geodesic structures (CLANs)^43^,^44^,^45^,^46^,^47^. With the exception of CLANs, which may be transient, our actin findings agree with these observations. Our tissue-based actin observations are unique, however, as they were made *in situ* in unsectioned TM retaining its original 3D configuration. This was possible by TPEF that penetrates deeper and causes less phototoxicity than conventional 1-photon microscopy.

We only used dye-based tissue labeling. Live-dead intravital dye co-labeling^34^,^41^ was used to determine if Lat-A adversely affected viability as the actin cytoskeleton has important roles in cell function. We did not see significantly reduced tissue viability following 1 μM Lat-A exposure over 2 days. Lat-A did not change autofluorescence intensity, which in the TM originates mainly from fibrillar collagen and elastin^40^,^42^. The potential for toxicity over longer exposure periods remains to be determined, however. Other promising cell-directed therapies affecting actomyosin activity, such as Rho kinase inhibitors, may be tested by our methods. These drugs are designed for long-term use in patients and hence their potential toxicity is important to characterize.

A secondary observation of our study was that Lat-A exposure was associated with larger, more interconnected TM pores. Delicate pores in the human TM retaining its original 3D configuration could be seen and measured by TPEF. Serial optical sections were captured through pores in specific tissue regions without artifacts of physical sectioning. Furthermore, the TPEF autofluorescence signature allowed for standardized identification of pore borders with ECM without contribution of cells. These characteristics are critical to capturing TM pore morphology quantitatively and in a way that is standardized and without confounding artifacts of traditional histologic sectioning (e.g., variable cutting vectors, tissue distortion, loss of 3D structure, lower imaging resolution). This permitted observations that were true to the 3D configuration of the tissue, which would have been hard to achieve by conventional histologic techniques. Our weak IHC staining pattern for type I collagen agreed with previous reports^27^,^31^.

We speculate that the TM’s elastic ECM yields to the contractile tone of cells attached to the structural ECM. This contractile tone is determined by actomyosin activity that in turn modulates tissue structure and pore configuration. In non-human primates, pharmacologically-induced cellular relaxation expands the drainage tissue and markedly reduces drainage resistance^24^. Our observations in human TM are a corollary to these non-human primate observations, suggesting active cellular mechanisms common to monkeys and humans by which outflow resistance and IOP are regulated at the TM level. Contractility intrinsic to the TM likely accounted for these pore changes as the ciliary muscle was disinserted in our tissue-based model.

Ancillary biomechanics studies are now needed to explore the role of ECM compliance in pore responses to contractile state. It is also worth determining if actomyosin-mediated pore expansion affects tissue preferential outflow. These important questions lie outside the scope of our study, which focused primarily on analyzing actin polymerization as a readout for TM contractility, and secondarily on whether contractile state affects tissue ECM configuration.

Our approach exploits technological advances in biological imaging^35^,^36^,^37^,^38^,^39^,^48^,^49^ and image analysis to bridge the gap between traditional cellular analysis *in vitro* and human tissue models, providing a unique translational platform. The platform may be used to quantify dose-dependency of effect and toxicity to guide dosing strategies. Other important effects of cell manipulation such as altered protein expression may also be analyzed, as we have shown *in situ*^33^.

Our tissue-based approaches and observations provide insights into actomyosin activity and ECM responses in the human TM. They are pertinent to understanding IOP regulatory mechanisms and pre-clinical development of novel cell-directed glaucoma therapy. These approaches may be adapted to test diverse therapies that have broader application beyond glaucoma.

## Materials and Methods

### Ethics Statement

Experiments and procurement of human donor corneoscleral tissue were approved by the Health Sciences Institutional Review Board at the University of Southern California and complied with the Declaration of Helsinki. Original cadaver corneal tissue was procured through the Doheny Eye Bank (Los Angeles, CA, USA) with written consent from the next of kin.

### Reagents

Low glucose DME, L-glutamine, amphotericin B, and DMSO (to dissolve calcein AM) were purchased from Mediatech (Washington, D.C.). Penicillin/streptomycin was purchased from the Norris Comprehensive Cancer Center Cell Culture Core (Los Angeles, CA). Serum-free media comprising low glucose DME, streptomycin, penicillin, gentamicin and L-glutamine were used. Alexa Fluor© 568-conjugated phalloidin and Hoechst 33342 were purchased from Thermo Fisher Scientific (Waltham, MA). Latrunculin-A (Lat-A) was purchased from LKT Laboratories, Inc. (St. Paul, MN).

### Human Donor Tissue Preparation

#### Human donor corneoscleral rim tissue

The tissue was generously provided by physicians of the Doheny Corneal Service (8 unique donors). Tissue was typically transplanted within 6 days postmortem (Oral communication, Dr. Martin Heur, August 19, 2011), received immediately after surgery, and maintained in Optisol GS transport medium (Bausch & Lomb, Rochester, NY) at 4 °C, as previously described^32^.

### Viability

Sample tissue wedges from each donor were labeled with viability-determining intravital dyes and analyzed as previously described^32^,^33^,^34^,^41^. Wedges were co-incubated with 0.3 μM calcein AM, a cytosolic vitality dye, and 1 μg/mL propidium iodide (PI)^50^,^51^, which labels necrotic and apoptotic nuclei, in phosphate-buffered saline (PBS) (1X, pH 7.4) for 30 min at 37 °C and 8% CO_2_. Some wedges were incubated with 0.2% Triton X-100 in PBS for 30 min prior to labeling to serve as dead controls. TPEF optical sectioning was performed through the entire TM. Calcein-positive cells and PI-positive cells were counted through entire z-stacks. Viability was defined as the percent of calcein-positive cells relative to total cells (calcein-positive cells plus PI-positive cells). Tissue with viability below 50% was excluded.

### Treatment

For two-photon imaging, human donor corneoscleral tissue was prepared as follows. Tissue was placed with the TM-side up onto a 150 × 20 mm glass dish (VWR International, Radnor, PA) in 5 mL of Optisol GS. The tissue was cut into 30° wedges with a razor blade, washed twice with 2 mL PBS, and incubated with (a) 2 mL serum-free media alone, or (b) 2 mL serum-free media and 1 μM Lat-A for 2 d at 37 °C and 8% CO_2_.

For western blot and immunohistochemistry analysis of elastin and type I collagen in the HTM, rims were cut in half (two halves, 2 × 180°) and incubated with either serum-free media alone or with Lat-A, as described above.

### Western blots

The dissected TM stripes from 4–12 donor tissues were pooled and homogenized in in Pierce© RIPA buffer (Thermo Fisher Scientific) supplemented with protease inhibitors (AMRESCO Inc, Solon, OH) using a motor homogenizer (5 mm × 75  mm generator, PRO scientific Inc, Oxford, CT), done 8–10 pulse twice on ice. The lysates following 30 min incubation on ice were centrifuged at 17,934 × g (13,000 rpm) and 4 °C for 20 min. The supernatant was collected and measured for protein concentration by BCA analysis (Pierce© BCA Analysis, Thermo Fisher Scientific). Twenty to sixty microgram of total protein were separated using a 10% reducing, acrylamide gel and SDS-PAGE and transferred on polyvinylidene fluoride membrane (Bio-Rad Laboratories, Hercules, CA) using Trans-Blot Turbo transfer system (Bio-Rad Laboratories). The blots were blocking in 5% nonfat dry milk in TTBS (Bio-Rad Laboratories) for 1 hour at room temperature (RT) and incubated with primary antibodies overnight at 4 °C followed by secondary antibodies for 1 hour at RT. For primary antibodies, mouse monoclonal antibodies to the α1 subunit of type I collagen (Millipore, clone 5D8-G9; 1:500) or rabbit anti-collagen I (1:1000, Abcam), and rabbit anti-α-elastin (1:200, Elastin Products Company, Owensville, MO), mouse anti-β-actin (1:5,000, Sigma) for house keeping gene; for secondary antibodies, HRP-conjugated secondary goat antibodies to rabbit IgG and mouse IgG (Novex®, Thermo Fisher Scientific). The blots were visualized by an enhanced chemiluminescence kit (Amersham ECL Prime Western Reagent, GE Healthcare, Wauwatosa, WI) and a chemiluminescence imaging system (ChemiDoc XRS + , Bio-Rad). Densitometry was performed using ImageLab 5.2.1 (Bio-Rad).

### Immunohistochemistry

Donor corneoscleral rim wedges were fixed in 4% paraformaldehyde (PFA) overnight at 4 °C. Subsequently, the wedges were embedded in Tissue-Tek optimum cutting temperature compound. Cryosections (7 μm thickness) were post-fixed with 4% paraformaldehyde and further permeabilized/blocked in blocking solution (5% bovine serum albumin and 0.3% Triton X-100 in PBS) for 1 h at room temperature. Sections were incubated overnight at 4 °C with primary antibodies (anti-alpha1 subunit of type I collagen, MAB 1340, EMD Millipore, Billerica, MA; rabbit polyclonal anti-alpha elastin, ab21607, Abcam, Cambridge, UK) in blocking solution. After washes with PBS the sections were further incubated for 1 h at room temperature with secondary, Alexa Fluor–conjugated anti-rabbit or mouse antibodies, as appropriate, and then mounted using ProLong Gold Anti-fade reagent with 4′,6-diamidino-2-phenylindole. Negative, nonspecific labeling was established with normal immunoglobulin G isotype controls. Sections were analyzed with a Keyence BZ-X700 fluorescence microscope (Itasca, IL). Immunofluorescence staining for each marker was performed using randomly selected slides (4–5 slides per each eye) containing four sections per slide and examined under the confocal microscope.

### Fixation and labeling for two-photon imaging

Tissue wedges were placed in 24-well plate wells, washed twice with 1 mL PBS, fixed for 30 min in 1 mL 4% PFA, permeabilized for 2 h in 1 mL of 5% Triton X-100 in PBS, rocking at 4 °C, and blocked in 1% BSA for 30 min at RT. Wedges were placed in 96-well plate wells and incubated with 150 μL of Alexa Fluor© 568-conjugated phalloidin and Hoechst 33342 in 0.1% BSA/PBS overnight (c.a. 16 h) at 4 °C on a rocking platform. They were then transferred to 24-well plates and washed 3X with 1 mL PBS for 15 min at RT.

### Two-photon Imaging

Tissue wedges were imaged with a Leica TCS SP5 AOBS MP confocal microscope system (Leica Microsystems, Heidelberg, Germany) coupled to a Chameleon Ultra-II multiphoton laser (Coherent, Santa Clara, CA). The wedges were placed TM-side down onto a glass-bottom microwell dish. Incident light was focused, and emitted signals were collected, with an inverted HCX PL APO CS 63X/1.3NA glycerol objective (Leica). The laser was centered at 850 nm to excite autofluorescence and Hoechst 33342-fluorescence. TPEF signals were collected in epifluorescence configuration, split with a dichroic mirror, passed through multiphoton bandpass filters (TPEF = 525/50 or 500–550 nm [Leica]; and red epifluorescence = 635/90 or 590–680 nm [Chroma, Bellows Falls, VT]) and guided onto a non-descanned photomultiplier tube detector (Hamamatsu, Bridgewater, NJ).

Tissue images were collected as series of optical sections (X, Y, Z; 600 Hz, bidirectional) using 1024 × 1024 pixel frames, 16-bit resolution and 16X line averaging. Images were captured using LAS AF (Leica) and analyzed with Volocity 5.4.1 (PerkinElmer; Waltham, MA), LAS AF Lite 2.2.1 (Leica), Imaris 7.3.0 (Bitplane; Zurich, Switzerland), and Image J. Images were cropped, resized and fit into figures using Photoshop CS5.

### Tissue image analysis

Qualitative observations of F-actin were made in the different regions of the TM (UM, CSM and JCT). Quantitative image analysis, however, focused on a 38 μm-thick CSM region located 25 μm from the inner uveal meshwork surface to allow for standardization. Here, in the specified CSM region, a fixed criterion could be applied to measurements made across different tissues.

#### (a) Qualitative analysis of F-actin organization

F-actin organization was qualitatively assessed in 2D optical sections through the UM, CSM and JCT and in 3D tissue reconstructions. Optical sections were loaded onto Leica LAS AF Lite and the UM, CSM and JCT were located using autofluorescence (AF) landmarks based on our prior characterization^32^.

#### (b) Quantitative analysis of F-actin density in 2D optical sections

Density was determined by averaging F-actin fluorescence intensity over serial optical sections 2 μm apart^52^, within 3D reconstructions of the CSM, and adjusting for nuclear counts in serial optical sections 8 μm apart to account for variation in cellular distribution in image samplings. Five same-sized rectangular ROI (3,230 μm^2^; Supplementary Fig. 1) were placed within each optical section. ROI position in an image section was maintained across other images in a series. Total ROI area was constant across tissues. F-actin fluorescence intensity based on mean gray values in each ROI in each optical section was recorded then averaged across corresponding ROIs in subsequent optical sections making up an image series. To adjust for variation in cellular distribution, F-actin mean fluorescence intensity in an ROI was divided by nuclear count in the ROI (see below, “Nuclear counting”; Supplementary Fig. 2) to give an F-actin intensity-to-nuclear ratio. F-actin intensity-to-nuclear ratios averaged across ROIs provided F-actin adjusted fluorescence intensity; this represented F-actin density adjusted for nuclear distribution (equation 1).


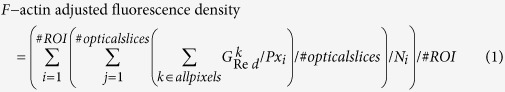


Here, *Gkred* is the gray scale (fluorescence intensity) value for each pixel (*k*) in the red emission fluorescence channel. *Px* is the number of pixels in the ROI (*i*). *N* is number of nuclei counted for each individual ROI. Paired, one-way Student’s t-testing was used to compare F-actin adjusted fluorescence intensity in vehicle control and Lat-A-treated tissue. A log transformation was used to establish a normal distribution. p-value of ≤0.05 was considered significant.

#### (c) Quantitative analysis of F-actin 3D distribution

F-actin 3D tissue distribution was measured in isosurface maps^53^ rendering the volume of F-actin above a predetermined threshold of size and red fluorescence intensity. This was performed in reconstructed CSM tissue volumes (246 × 246 × 38 μm-thick; Supplementary Fig. 1C). Optimum threshold parameters were determined in separate testing as: (a) voxels of 0.24 × 0.24 × 1 μm in X, Y, Z axes, respectively. This captured cortical F-actin and coarse punctate F-actin features with dimensions ≥0.24 μm; (b) fluorescence intensity cutoff greater than 10,025 but less than 65,540 per voxel, based on 16-bit resolution.

Resulting isosurface maps provided reasonable re-creations of F-actin distribution in the tissue. Each reconstructed tissue volume was further segmented into nine equal-sized subvolumes (82 × 82 × 38 μm in X, Y, Z axes, respectively; Supplementary Fig. 1 C) for quantitative analysis of isosurface map volumes. To adjust for variation in cellular distribution, F-actin mean map volume in each tissue subvolume (cuboid) was divided by number of nuclei in the subvolume (see next, “Nuclear counting”) to give an F-actin distribution-to-nuclear ratio. F-actin fluorescence distribution-to-nuclear ratios averaged across ROIs yielded an F-actin adjusted fluorescence distribution value (equation 2) representing F-actin distribution. Paired, one-way Student’s t-testing was used to compare F-actin distribution-to-nuclear ratios in vehicle control and Lat-A-treated tissue. A log transformation was used to establish a normal distribution. A p-value of ≤0.05 was considered significant.


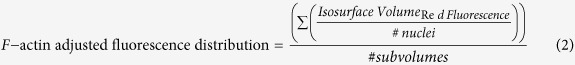


#### (d) Quantitative analysis of structural ECM configuration

Autofluorescence revealed pores among the multilayered plates of the CSM. The cross-sectional areas of distinct pores were calculated by first measuring the diameters of the short and long axes and then applying the following formula (equation 3) for elliptical area:


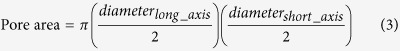


Four to twelve pores were measured per z-stack. Selected pores were limited to those that were entirely contained within the image frame, thus eliminating any pores that were artifactually sectioned.

The sum of all pore volumes contained within each z-stack was also determined, using a method similar to that described above (F-actin 3D distribution). The CSM was cropped in all three dimensions to avoid any excess signal voids. Then the AF signal was reconstructed as isosurface volumes using the following parameters: surface grain size of 2 μm, largest sphere diameter or 1.803 μm, fluorescence intensity minimum cutoff of 3,600 (adjusted per treatment pair, as required by variations in overall fluorescence intensity), and minimum voxel size of 10 μm. A solid block of fluorescence was then generated in software to serve as a mask (all voxels within the z-stack set to a value of 20,000) from which to carve out a negative imprint of the AF. The AF isosurface volume was then subtracted from the solid mask to generate a 3D representation of the pores. Isosurface volumes of the pores were reconstructed using the following parameters: surface grain size of 0.481 μm, largest sphere diameter of 1.803 μm, automatic (default) fluorescence intensity minimum cutoff value, and a minimum voxel size of 10 μm. Statistical testing for differences was by paired Student’s t-test.

#### (e) Quantitative analysis of autofluorescence intensity in 2D optical sections

Autofluorescence intensity was determined by utilizing the five same-sized rectangular ROI (3,230 μm^2^; Supplementary Fig. 1) established for the F-actin denstiy analysis described above (see Quantitative Tissue Analysis, b). Total ROI area was constant across tissues. Autofluorescence intensity based on mean gray values in each ROI in each optical section was recorded and mean autofluorescence intensity calculated for each donor and each condition. These values were pooled into the appropriate vehicle control or Lat-A-treated groups (n = 8). A paired Student’s t-test was performed

#### (f) Quantitative nuclear analysis

Nuclear counts allowed F-actin density and distribution measures to be adjusted for variation in cell distribution in image samplings. Optical sections 8 μm apart were captured in Imaris and transferred to Photoshop CS5. We had determined in separate studies that 8 μm intervals between optical sections were large enough to minimize nuclear double-counting, but small enough to avoid missing nuclei altogether, as illustrated in Supplementary Fig. 2^43^. All nuclei in ROI (for fluorescence intensity analysis for F-actin density) and image subvolumes (for fluorescence distribution analysis) were assigned a number in the software and counted.

#### (g) Quantitative toxicity analysis

Calcein-positive live cells were counted in 3D tissue reconstructions in Imaris either by a manual, frame-by-frame method or by a software-assisted, semi-automated method previously described^41^. Viability was expressed as the percentage of calcein-positive cells to all cells (i.e., calcein-positive (live) cells plus PI-positive (dead) cells) in vehicle control and Lat-A-treated TM. Each treated sample was compared with a vehicle control sample from the same donor eye. Toxicity was analyzed for vehicle control and Lat-A-treated tissue (n = 4 donors). A two-proportion Z-test was used to determine statistical significance (p ≤ 0.05).

## Additional Information

**How to cite this article**: Gonzalez, J. M. *et al.* Tissue-based multiphoton analysis of actomyosin and structural responses in human trabecular meshwork. *Sci. Rep.* **6**, 21315; doi: 10.1038/srep21315 (2016).

## Supplementary Material

Supplementary Movie 1

Supplementary Movie 2

Supplementary Data
