# Supplementary material for: Tissue-based multiphoton analysis of actomyosin and structural responses in human trabecular meshwork
Source: Sci Rep. 2016 Feb 17;6:21315. doi: 10.1038/srep21315 (PMC4756353; doi:10.1038/srep21315)
Supplement: Supplementary Data [file srep21315-s3.doc]

**Title:**

**Tissue-based multiphoton analysis of actomyosin and structural responses in human trabecular meshwork**

Authors:
Jose M Gonzalez, Jr*, PhD

Minhee K Ko*, PhD
Andrew Pouw*, B.S.
James C. H. Tan*, MD, PhD

**Supplementary Figures & Movies:**


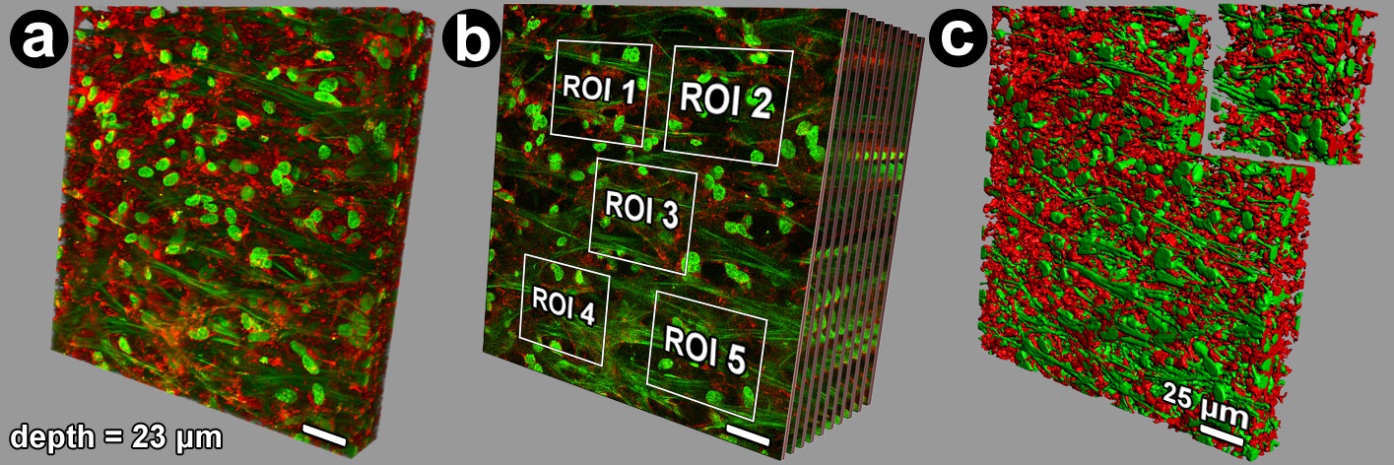


Supplementary Figure 1. Algorithms for measuring filamentous actin (F-actin) density and distribution in 3D reconstructions of human trabecular meshwork (TM). a: Phalloidin (red) amongst autofluorescent structure (green fibers) and Hoechst 33342-labeled nuclei (green ovals). Reconstruction is a 38 μm-thick section of the corneoscleral meshwork. b: F-actin density was analyzed in 5 randomly positioned same-sized regions of interest (ROI; white boxes) across 30 consecutive optical sections. c: F-actin distribution was analyzed in isosurface maps with volume renderings of the reconstruction data. Corneoscleral meshwork regions were segmented into nine same-sized floating cuboids (subvolumes). Depth from the inner uveal meshwork surface is indicated.


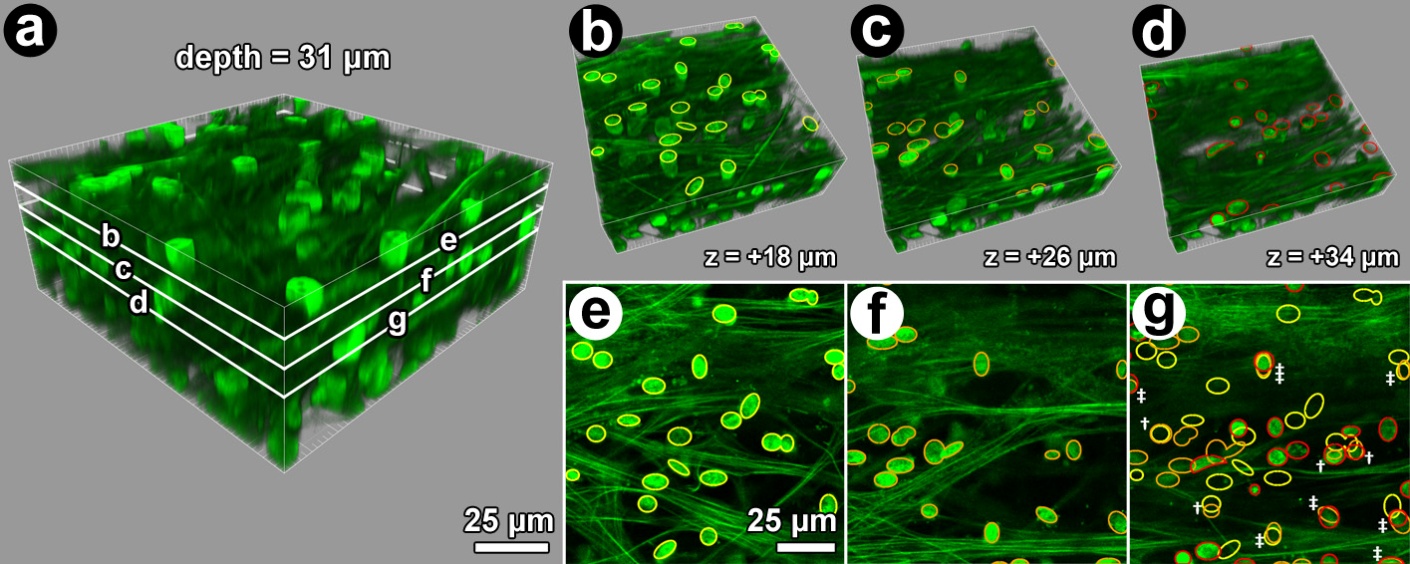


Supplementary Figure 2. Counting of Hoechst 33342-labeled nuclei amongst autofluorescent structure of corneoscleral meshwork. a: Rationale for performing cell counts in optical sections 8 μm apart, as illustrated by white lines. Line “b” intersects a Hoechst-labeled nucleus, but “c”, located 8 μm deep to ‘b’, lies beyond the nucleus. Lines “e”, “f”, and “g”, all 8 μm apart, illustrate that most nuclei have diameters approximating 8 μm or less in the z-axis. b-d: same tissue sliced to depths of 18 (b), 26 (c), and 34 μm (d) from the top surface of tissue reconstruction in a. e-g: optical sections at z-axis depths of 16 μm (e), 24 μm (f), and 32 μm (g) beyond starting depth of 31 microns from the uveal surface. Ovals/circles outlining nuclei were color-coded in different optical sections (e.g., yellow at +18 μm, orange at +26 μm, red at +34 μm), helping to avoid nuclear double-counting in serial optical sections. Depth from the inner uveal meshwork surface is indicated.


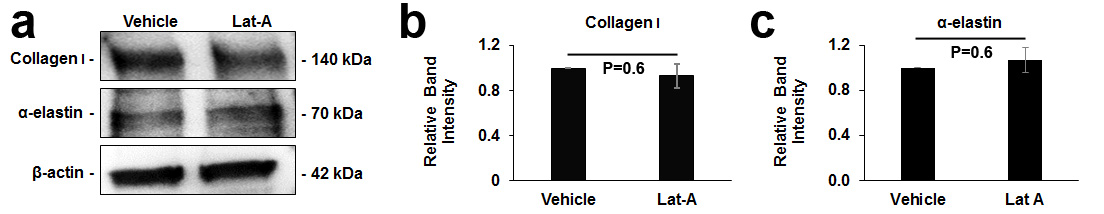


Supplementary Figure 3. Western blot and densitometry analysis of type I collagen (Collagen I) and elastin (α-elastin) in human TM with Lat-A treatment. a: Representative bands from two experiments pooling TMs from 4 and 12 donors after incubation for two days with DME alone (Vehicle) or DME and 1μM Lat-A. PVDF membranes were probed with antibodies for either the alpha 1 subunit of type I collagen, alpha-elastin, or beta-actin (as a loading control). Qualitatively the bands for collagen I seemed reduced, alpha-elastin increased, and beta-actin remaining the same with Lat-A treatment. b, c: However, densitometry revealed non-significant differences in the sizes of proteins bands between Vehicle and Lat-A treatment groups for Collagen I (b) and for α-elastin (c). Error bars = standard error of the mean.


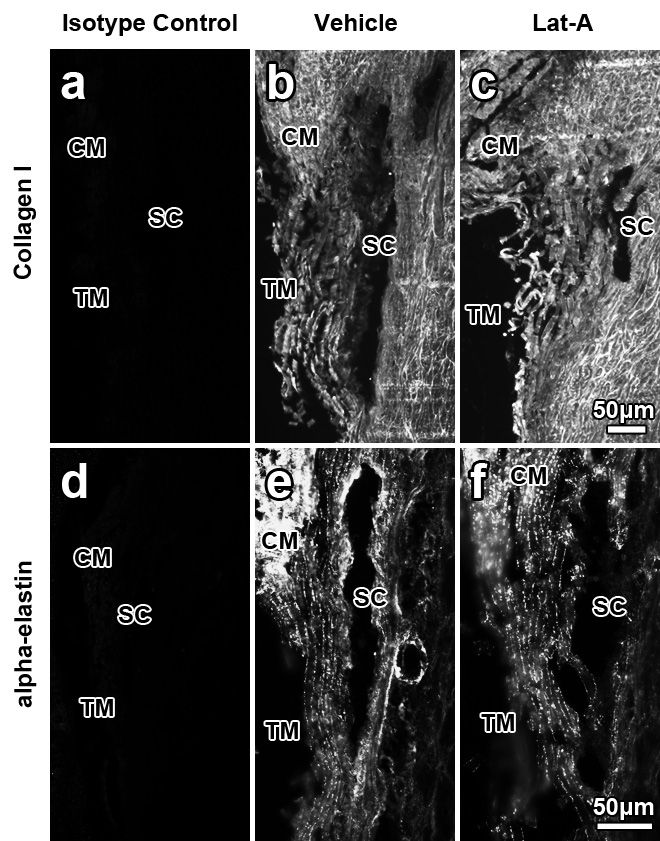


Supplementary Figure 4. Immunohistochemistry of human TM for type I collagen (Collagen I) and elastin (α-elastin) with Lat-A treatment. After treatment with DME alone (vehicle) or with DME and 1μM Lat-A, human TM was frozen, post-fixed, sectioned in the sagittal plane and incubated with mouse or rabbit IgG isotype controls (a, d, respectively) or with antibodies to either the alpha 1 subunit of type I collagen (b, c) or to alpha-elastin (e, f) and with the appropriate Alexa dye-conjugated secondary antibody. Staining intensities and distributions for either protein target were unchanged with Lat-A treatment relative to vehicle controls. CM: ciliary muscle. TM: trabecular meshwork. SC: Schlemm’s canal.

Supplementary Movie 1. 3D reconstruction of F-actin (red), autofluorescence (green structure), and Hoechst 33342-labeled nuclei (green ovals) in control human tissue, as depicted in Fig. 3A-C. The trabecular meshwork is viewed from the uveal and corneoscleral meshwork aspect. A prominent cortical F-actin network associates with autofluorescent structures. Punctate F-actin collections are seen.

Supplementary Movie 2. 3D reconstruction of F-actin (red), autofluorescence (green structure), and Hoechst 33342-labeled nuclei (green ovals) in human tissue treated with 1 µM Lat-A, as depicted in Fig. 3D-F. The trabecular meshwork is viewed from the uveal and corneoscleral meshwork aspect. A prominent cortical F-actin network is no longer seen but punctate collections prevail.
